# The Requirement of Sox2 for the Spinal Cord Motor Neuron Development of Zebrafish

**DOI:** 10.3389/fnmol.2020.00034

**Published:** 2020-03-27

**Authors:** Jie Gong, Songqun Hu, Zigang Huang, Yuebo Hu, Xiaoning Wang, Jinxiang Zhao, Peipei Qian, Cheng Wang, Jiajing Sheng, Xiaofeng Lu, Guanyun Wei, Dong Liu

**Affiliations:** ^1^School of Life Science, Nantong University, Nantong, China; ^2^Department of Otorhinolaryngology Head and Neck Surgery, Affiliated Hospital of Nantong University, Nantong, China; ^3^Key Laboratory of Neuroregeneration of Jiangsu and MOE, Co-innovation Center of Neuroregeneration, Nantong University, Nantong, China

**Keywords:** Sox2, motor neuron, axons, zebrafish, differentiation, development

## Abstract

Sex-determining region Y box 2 (Sox2), expressed in neural tissues, plays an important role as a transcription factor not only in the pluripotency and proliferation of neuronal cells but also in the opposite function of cell differentiation. Nevertheless, how Sox2 is linked to motor neuron development remains unknown. Here, we showed that Sox2 was localized in the motor neurons of spinal cord by *in situ* hybridization and cell separation, which acted as a positive regulator of motor neuron development. The deficiency of Sox2 in zebrafish larvae resulted in abnormal PMN development, including truncated but excessively branched CaP axons, loss of MiP, and increase of undifferentiated neuron cells. Importantly, transcriptome analysis showed that Sox2-depleted embryos caused many neurogenesis, axonogenesis, axon guidance, and differentiation-related gene expression changes, which further support the vital function of Sox2 in motor neuron development. Taken together, these data indicate that Sox2 plays a crucial role in the motor neuron development by regulating neuron differentiation and morphology of neuron axons.

## Introduction

Motor neuron disease (MND) characterized by muscle weakness or spastic paralysis is a neurodegenerative disease caused by progressive paralysis and death of motor stem neurons in the brainstem or spinal cord (Babin et al., 2014). Two different kinds of MNDs have been extensively studied. Spinal muscular atrophy (SMA), a hereditary MND that occurs in childhood, is characterized by selective loss of spinal motor neurons. The other is amyotrophic lateral sclerosis (ALS), an adult-onset neurodegenerative disease that specifically causes the degeneration of upper and lower motor neurons with the progressive weakness of muscle (Haramati et al., 2010). Elucidating the mechanisms involved in motor neuron development may variegate therapeutic strategies for MND.

Zebrafish is an excellent model system for studying human disease, especially neurobiological related diseases, due to its well-characterized embryo development and the labeled neurons (McWhorter et al., 2003). Motor neurons have precise subtype identities that are characterized by different morphological criteria and extend their axons to different target muscles in a highly specific manner (Shirasaki and Pfaff, 2002; Lewis and Eisen, 2003). In zebrafish, there are two different types of spinal motor neurons: primary motor neurons (PMNs) and secondary motor neurons (SMNs), in view of their formation time and target musculature (Myers et al., 1986; Babin et al., 2014). PMNs, localized relatively dorsally with large somatas and thick axons, can be further divided into three or four different categories in each spinal hemisegment. There are middle primary motor neurons (MiP), rostral primary motor neurons (RoP), caudal primary motor neurons (CaP), and variable primary motor neurons (VaP; Myers, 1985; Moreno and Ribera, 2009). The first three PMNs that are investigated extensively innervate the dorsal, middle, and ventral trunk musculature, respectively, while the VaPs that only exist in about half somitic hemisegments of zebrafish usually innervate an exclusive muscle fiber territory between RoP and MiP, and most of them undergo apoptosis during embryonic development between 20 and 36 hpf (Eisen et al., 1990). Different from the PMNs, SMNs are localized more ventrally in the motor column with typically smaller somatas and thinner axons, which are born 5–6 h later than PMNs (Myers et al., 1986). In most cases, each somitic hemisegment of zebrafish contains only one CaP, MiP, and RoP, but almost 25 SMNs that have the similar axonal pathfinding to PMNs (Zelenchuk and Brusés, 2011).

In vertebrates, the development of motor neurons and their axon trajectories has been extensively investigated and is known to be regulated by a series of genes through different dorsal or ventral expression profiles. It has been reported that, in mouse, sema3a and netrin1 could specifically inhibit dorsal axon extension (Messersmith et al., 1995). In addition, the lack and overexpression of EphA4 caused deviant trajectories of motor neuron axons by representatively preventing dorsal motor neurons from correctly projecting their axons into the target dorsal lamb muscles (Helmbacher et al., 2000). In zebrafish, Nkx6.1 and olig2 are expressed in the spinal cord and are necessary for motor neuron development. Missing Nkx6.1 may cause MiP axons to be truncated and excessively branched (Park et al., 2002; Hutchinson et al., 2007). Similarly, the dysfunctionality of survival motor neuron genes results in significant axon pathfinding defects. Without these genes, CaP axons can neither accurately project into the ventral muscles in terms of time nor fully enter the ventral muscles (McWhorter et al., 2003; Rodino-Klapac and Beattie, 2004). In our previous studies, we also found that insm1a and kinesin-12 play vital roles in the development of motor neurons (Xu et al., 2014; Gong et al., 2017). For example, insm1a mutant zebrafish have significant PMN defects, including reduction of CaPs and MiPs, excessively branched CaP axons, and abnormal distances between adjacent CaPs (Gong et al., 2017).

Sex-determining region Y box 2 (Sox2) is a transcription factor with a high-mobility-group type (HMG) domain, which belongs to the Sox B1 family together with Sox1 and Sox3 (Kamachi et al., 2000; Avilion et al., 2003). Sox2 is well known for its function in maintaining pluripotent properties of stem cells and reprogramming differentiated cells into stem cells (Graham et al., 2003; Takahashi and Yamanaka, 2006). In chicken embryos, transfection of Sox2 expression vectors into neural progenitors could inhibit their differentiation by maintaining the progenitor characteristics, while the deficiency of Sox2 promotes the separation of neural progenitor cells from the ventricular zone to exit from the cell cycle, indicating the inhibitory role of Sox2 on the differentiation of neural stem/progenitor cells (NSPCs; Graham et al., 2003). However, with further research, more and more studies show that Sox2 can positively regulate the neuronal differentiation. Recent studies have shown that Sox2 is expressed at low levels in mouse NSPCs. However, in the newly differentiated oligodendrocytes of the developmental myelination, Sox2 has remarkable expression, which indicates the necessary role of Sox2 in the differentiation and regeneration of oligodendrocyte (Pedre et al., 2011; Dai et al., 2015; Zhang et al., 2018a). Moreover, it has been deduced that Sox2 is indispensable for the development of sensory nerve in the inner ear of both mice and zebrafish to regulate hair cell regeneration or induce the volume of non-sensory neuron (Millimaki et al., 2010; Puligilla et al., 2010; Steevens et al., 2017; Gou et al., 2018). However, little research on the role of Sox2 in motor nerves has been done. Therefore, studying the function of Sox2 in the development of zebrafish embryonic motor neuron can provide new insights into the roles of Sox2 in the development of vertebrate neurons. In this study, we first detected the expression of Sox2 in zebrafish and verified its localization in the spinal cord and motor neurons, and then we examined the function of Sox2 in PMN development by both knockdown and knockout techniques in *Tg(mnx1:GFP)^ml2^* transgenic zebrafish and investigated the possible mechanism during this process.

## Materials and Methods

### Zebrafish Line

The zebrafish embryos and adults were maintained in the zebrafish Center of Nantong University under conditions in accordance with our previous protocols (Wang et al., 2016). The transgenic zebrafish line *Tg(mnx1:GFP)^ml2^* has been described in a previous work (Gong et al., 2017).

### Cell Separation, RNA Isolation, Reverse Transcription, and Quantitative RT-PCR

A total of 300–400 *Tg(mnx1:GFP)* zebrafish embryos were prepared by dechorionation at 24 hpf and washed three times with PBST and then three times with Ca^2+^ free Ringer’s solution. After being trypsinized by 0.25% trypsin, the reaction was terminated by 10% FBS and filtered through 100- and 40-μm filter membranes. The samples were analyzed using a flow cytometer (BD, Franklin Lakes, NJ, USA). The cells with GPF fluorescence were identified as positive cells.

Total RNA was extracted from zebrafish embryos and the cells were separated *via* a flow cytometer by TRIzol reagent according to the manufacturer’s instructions (Invitrogen, Waltham, MA, USA). Genomic contaminations were removed by DNaseI, and then 2 μg of total RNA was reversely transcribed using a reversed first-strand cDNA synthesis kit (Fermentas, Waltham, MA, USA) and stored at −20°C. Quantitative RT-PCR was performed using the corresponding primers (Supplementary Table S1) in a 20-μl reaction volume with 10 μl of SYBR premix (Takara, Japan) and *elongation factor 1a* (*ef1a*) was used as the internal control. All samples were analyzed in triplicate.

### Whole Mount *in situ* Hybridization

A 428-bp cDNA fragment of Sox2 was amplified from wild-type embryo cDNA using the specific primers of Sox2 F1 and R1 (Supplementary Table S1). Digoxigenin-labeled sense and antisense probes were synthesized using linearized pGEM-T-easy vector subcloned with this Sox2 fragment by *in vitro* transcription with DIG-RNA labeling kit (Roche, Switzerland). Zebrafish embryos without pigment at different developmental stages were collected and fixed with 4% PFA overnight, and then whole mount *in situ* hybridization (WISH) was performed as described in the previous study (Huang et al., 2013). For sectioning, the whole-mount *in situ* hybridized embryos stored in 100% glycerol were transferred to Tissue-Tek OCT compound before finally being embedded in OCT blocks. Then, the blocks were trimmed and sectioned on a Leica RM2125 microtome at 12 μm. After 4 h drying at 37°C, the sections were washed three times with PBS and then mounted with the mounting medium.

### Morpholino, mRNAs, and Construct Injections

Translation-blocking morpholino (5′-GCTCGGTTTCCATCATGTTATACAT-3′) against the ATG-containing sequence was synthesized by Gene Tools. Morpholino was diluted to 0.3 mM with RNase-free water and injected into one-cell stage embryos and then raised in E3 medium at 28.5°C for imaging.

The Sox2 cDNAs containing the open reading frame were cloned into PCS2^+^ vector. After this recombinant plasmid was linearized, the Sox2 mRNA was synthesized *in vitro* by the mMESSAGE mMACHIN Kit (Ambion, USA) according to the manufacturer’s instruction, and then the capped mRNAs were purified using RNeasy Mini Kit (Qiagen, Hilden, Germany). Two nanoliters of Sox2 mRNA was injected at 20 ng/μl into one-cell stage embryos.

The Sox2 ORF was cloned from the zebrafish by PCR, and then cloned into a pME-MCS vector to produce middle entry clone (pME-Sox2). p5E-mnx1 plasmid was obtained from Addgene. To generate an expression construct, p5E-mnx1, pME-Sox2, and p3E-polyA were combined with pDestTol2pA2 by the LR recombination reaction as described in the Lifetech Multiste Gateway Manual (Figure 5E; Life Technologies, Carlsbad, CA, USA). Subsequently, this construct was co-injected with tol2-transposase mRNA into one-cell stage Sox2 mutant zebrafish embryos to create the mosaic rescue zebrafish model.

**Figure 1:**
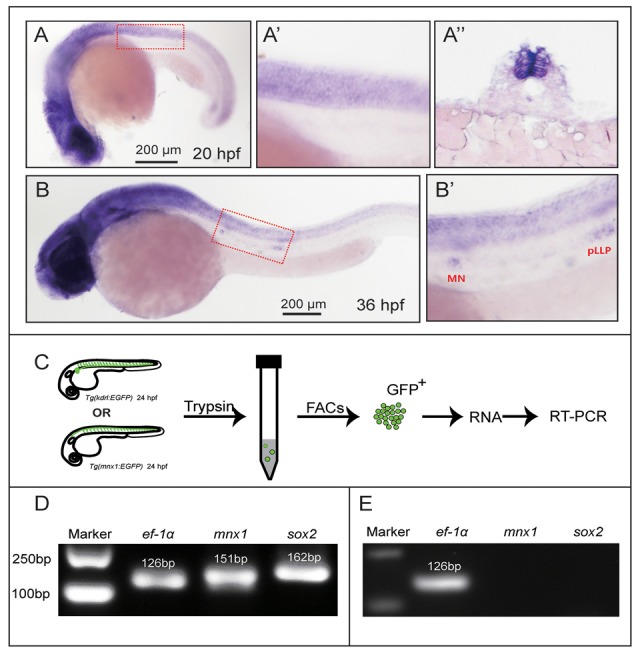
Sex-determining region Y box 2 (Sox2) expression analyses in the spinal cord and motor neurons. **(A)** At 20 hpf, the *in situ* hybridization signal of Sox2 is localized in the spinal cord. **(A′)** The magnified figure of the region squared in dashed line. **(A″)** The trunk transverse section of the embryos. **(B)** Sox2 is expressed in the spinal cord and the deposited neuromasts. **(B′)** The magnified figure of the region squared in dashed line. Scale bar = 200 μm. **(C)** The procedure of the motor neurons and endothelial cells sorting and RT-PCR. **(D)** The results of the RT-PCR on Mnx1-GFP sorted cells. Sox2 is expressed in the selected neuron cells from the *Tg(Mnx1:EGFP)* line. **(E)** The result of the RT-PCR on Kdrl-EGFP sorted cells. No signals of Sox2 and mnx1 are detected in the GFP-positive cells sorted from the *Tg(kdrl:EGFP)* line.

**Figure 2:**
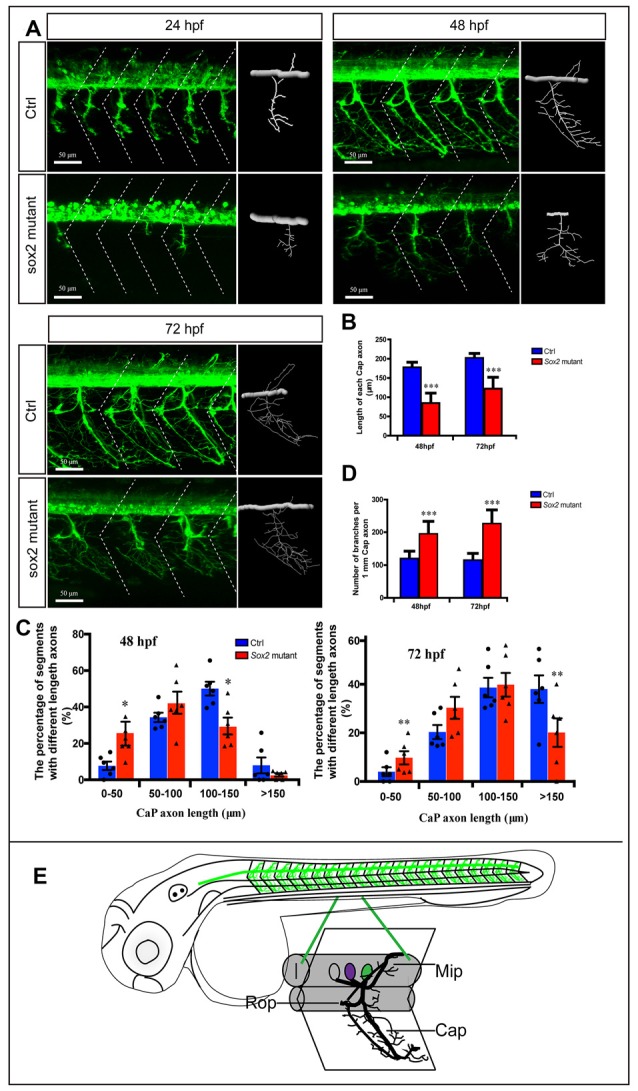
Primary motor neuron morphogenesis defects in the Sox2 knockout zebrafish. **(A)** Confocal imaging analysis of primary motor neurons in control and Sox2 knockout groups at 24, 48, and 72 hpf. The dotted diagonal line marked the boundary of the myosepta. Scale bar = 50 μm. **(B)** The length of CaP axons in control and Sox2 knockout zebrafish at 48 hpf (*n* = 21 and 33, respectively) and 72 hpf (*n* = 13 and 29, respectively). **(C)** The variability of CaP phenotype across segments in controls and Sox2 mutants at 48 and 72 hpf. Circle means control and triangle means Sox2 mutant. **(D)** The number of branches per 1-mm CaP axon in control and Sox2 knockout zebrafish at 48 hpf (*n* = 11 and 9, respectively) and 72 hpf (*n* = 8 and 12, respectively). Each bar represents the mean ± SD. Values with *, **, and *** above the bars are significantly different (*P* < 0.05, *P* < 0.01, and *P* < 0.001, respectively). **(E)** The schematic for three different primary motoneurons (CaP, MiP, and RoP) in the whole fish and a specific myotome segment.

**Figure 3:**
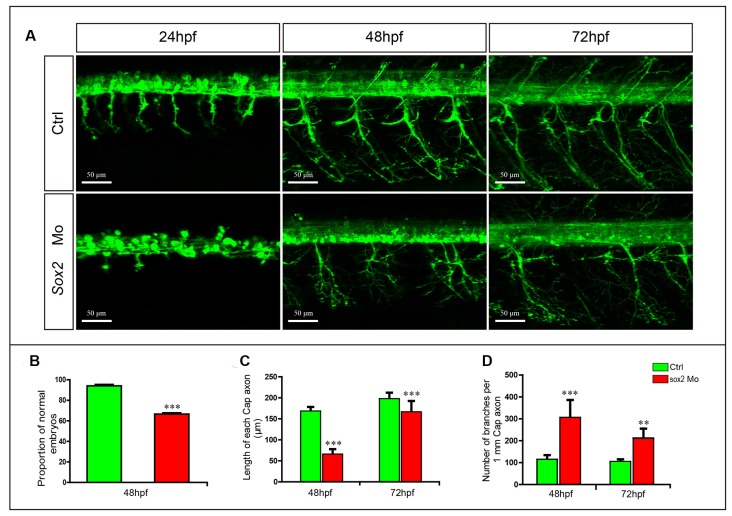
Primary motor neuron developmental defects in the Sox2 morphants. **(A)** Confocal imaging analysis of primary motor neurons in wild type and Sox2 morphants at 24, 48, and 72 hpf. Scale bar = 50 μm. **(B)** Quantification of zebrafish embryos with abnormal PMNs (*n* = 176 and 238, respectively). **(C)** The length of CaP axons in control and Sox2 morphants at 48 hpf (*n* = 21 and 32, respectively) and 72 hpf (*n* = 17 and 19, respectively). **(D)** The number of branches per 1-mm CaP axon in control and Sox2 morphants at 48 (*n* = 8 and 7, respectively) and 72 (*n* = 5 and 6, respectively) hpf. Each bar represents the mean ± SD. Values with ** and *** above the bars are significantly different (*P* < 0.01 and *P* < 0.001, respectively).

**Figure 4:**
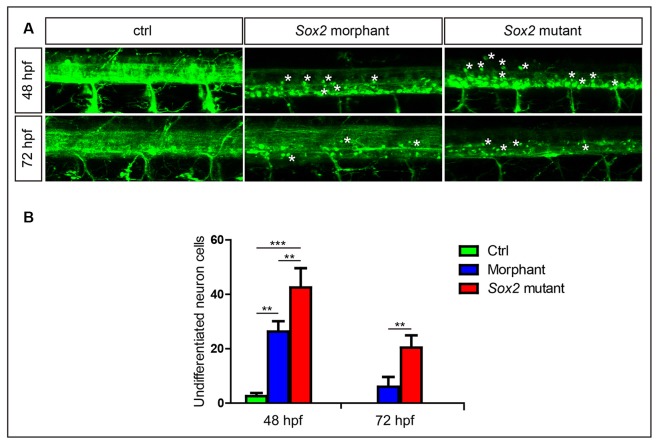
Sox2 deficiency suppressed neuronal cell differentiation. **(A)** Confocal imaging analysis of primary motor neurons in the control group, Sox2 mutant group, and morphant group at 48 and 72 hpf (*n* = 4). Asterisks indicate undifferentiated neuronal cells. **(B)** Quantification of the undifferentiated neuronal cell in the three different groups. Each bar represents the mean ± SD. Values with ** and *** above the bars are significantly different (*P* < 0.01 and *P* < 0.001, respectively).

**Figure 5:**
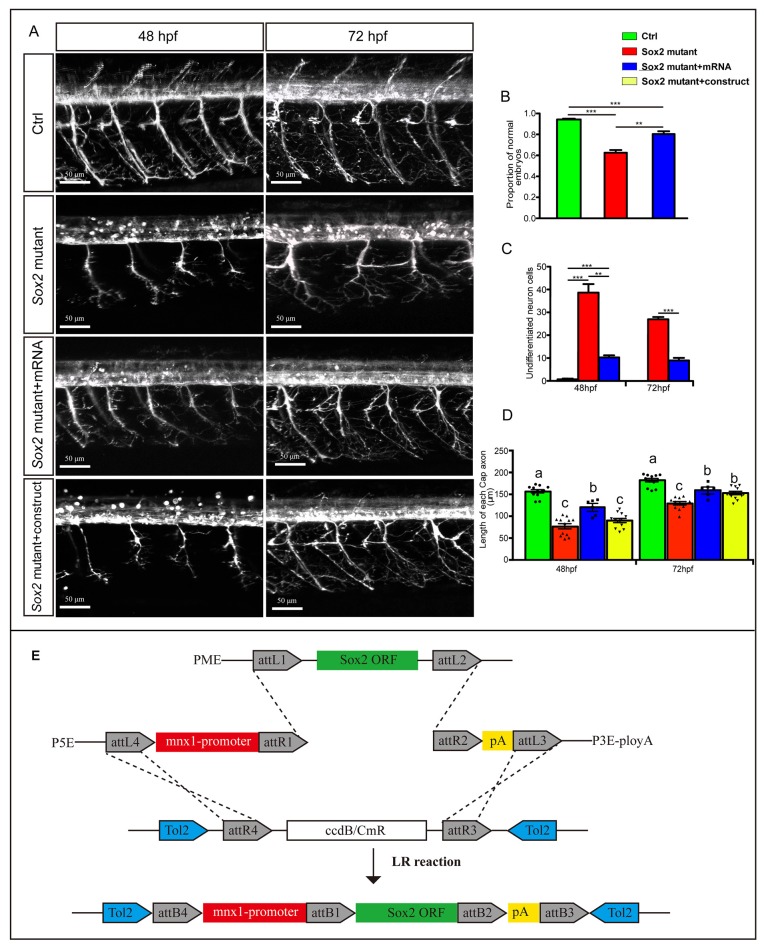
Overexpressions of Sox2 rescued the motor neuron defects in Sox2 mutant embryos. **(A)** Confocal imaging analysis of primary motor neurons in three different groups at 48 and 72 hpf. Scale bar = 50 μm. **(B)** Quantification of zebrafish embryos with abnormal PMNs (*n* = 120, 241, and 132, respectively). **(C)** Quantification of the undifferentiated neuronal cells in the three different groups (*n* = 4). Values with ** and *** above the bars are significantly different (*P* < 0.01 and *P* < 0.001, respectively). **(D)** Quantification of the length of CaP axons in the four different groups (*n* = 12 and 6). Circle means control, triangle means Sox2 mutant, square means Sox2 mutant +mRNA, and inverted triangle means Sox2 mutant +construct. Values with different letters on the top of bars are significantly different (*P* < 0.05). **(E)** Multisite Gateway cloning (three-way multisite reaction) to generate a motor neuron specific Tol2 construct for expression of Sox2 mRNA.

### TUNEL Assay

For TUNEL assays, zebrafish embryos at 24 hpf were fixed in 4% PFA at 4°C overnight. After being rinsed with PBST for 10 min, the embryos were incubated in proteinase K for 2 min, followed by two PBST washes for 5 min each time. Then, the embryos were refixed with 4% PFA for 10 min. After being rinsed with PBST three times for 5 min each, the tunnel assay was carried out using the Cell Death Detection Kit (Roche, Nutlet, NJ, USA) following the manufacturer’s directions.

### cDNA Library Preparation and RNA Sequencing

Total RNA was extracted from the Sox2 mutant and wild-type zebrafish at 32 and 48 hpf, respectively, using TRIzol Reagent (Invitrogen, Waltham, MA, USA). Subsequently, the integrity and purity were estimated by NanoDrop 2000 (Thermo Fisher Scientific Inc., Waltham, MA, USA). Only high-quality RNA samples (OD260/280 ranged 1.8–2.2, RIN ≥ 8.0) were used to construct the sequencing library. Two pooled RNA samples at each time point and group were prepared for sequencing. The final sequencing cDNA libraries were quantified and sequenced using the Illumina HiSeq 4000 with 150-bp pair-end reads produced (Illumina, San Diego, CA, USA).

### Microscopy and Statistical Analysis

After being anesthetized with tricaine, the zebrafish embryos were mounted in 0.8% low melt agarose, and then photographed using a Leica TCS-SP5 LSM confocal microscope. For the quantitation of the length of CaP axons and CaP branches, we imported the images into imaris software and traced CaP axons and all the branches. Then, we quantified the length of CaP axons and the branch points. For the *in situ* hybridization, photographs were taken using an Olympus stereomicroscope MVX10. Statistical analyses were performed by one-way analysis of variance (ANOVA) or Student’s *t*-test, and *P*-values < 0.05 were considered statistically significant.

## Results

### Sox2 Was Expressed in Spinal Cord and PMNs of Zebrafish

Firstly, we tested the expression profile of Sox2 in zebrafish by WISH with a digoxigenin-labeled Sox2 probe. The results showed that Sox2 was expressed in the brain and spinal cord at 20 and 36 hpf (Figures 1A,B). In addition, the localization of Sox2 in the spinal cord was further verified by transverse section analysis (Figure 1A″). When the embryo developed to 36 hpf, Sox2 was also localized in both the migrating posterior lateral line primordium and the deposited neuromasts (Figure 1B′).

In order to further analyze the expression level of Sox2 in the zebrafish nervous system and whether it was expressed in the motor neurons, we selected the motor neurons from *Tg(mnx1:GFP)^ml2^* whose motor neurons were labeled by GFP for the RNA extraction. The results of RT-PCR showed that both mnx1 and Sox2 were detected in the selected neuron cells (Figure 1D), which indicated that Sox2 might be expressed in zebrafish motor neurons. Moreover, we performed RT-PCRs on Sox2-negative tissue. The results showed that no Sox2 and mnx1 signal were detected in the GFP-positive cells sorted from the *Tg(kdrl:EGFP)* line (Figure 1E), in which endothelial cells were labeled with GFP.

### Deficiency of Sox2 Caused Developmental Defect of PMNs

In order to investigate the function of Sox2 during the motor neuron development, we examined the morphology of PMNs in the Sox2 knockout *Tg(mnx1:GFP)^ml2^* transgenic zebrafish. Four different types of mutations, an 11-bp deletion, a 4-bp deletion, a 3-bp deletion, and a 3-bp insertion, were identified in the F1 zebrafish (Supplementary Figure S1). These lines shared similar phenotypes and the 4-bp deletion mutant line that translated a significantly truncated protein was used for the following experiments.

The morphology of PMNs was examined by confocal microscopy at 24, 48, and 72 hpf, which showed significant developmental defects in the Sox2 mutant fish (Figure 2). Compared with the control group, the development of CaPs was significantly inhibited. For example, the CaPs of the control fish at 24 hpf almost projected their axons to the horizontal myoseptum, while most of the CaPs in Sox2 mutants did not generate the axons or shortened the axons (Figure 2A). In addition, the length of main CaP axons was measured at 48 and 72 hpf and the results showed that at 48 hpf, the average axon length of CaP in the Sox2 mutants was 84 ± 26 μm, which was significantly shorter than that of the control group (177 ± 13 μm). When developed to 72 hpf, although the axons of CaP in the mutants obviously grew, they were still shorter than the control (122 ± 29 μm vs. 202 ± 11 μm), indicating that the truncated axons could not recover completely (Figures 2A,B). Moreover, we also measured variability of CaP phenotype across segments in controls and Sox2 mutants at 48 and 72 hpf by imaging the whole trunk of zebrafish, which would clearly reveal the phenotype caused by the deficiency of Sox2. The results showed that the percentage of segments with short axons in the Sox2 mutant zebrafish was significantly higher than that in the control fish, while ratio of long CaP axons in the Sox2 mutant zebrafish was significantly fewer than that in the control fish (Figure 2C).

In addition, the number of CaP branches of Sox2 mutants also significantly increased at 48 and 72 hpf (Figures 2A,D). Beside CaP, the other two kinds of motor neurons, MiP and RoP, were also affected by the lack of Sox2, such as changes in axon morphology or even reduction in the number of MiP (Figure 2A and Supplementary Figure S2). To further confirm that the motor neuron changes were specifically caused by Sox2 inactivation, Sox2 translation blocking morpholino was injected into zebrafish embryos. Sox2 morphants showed the similar motor neuron morphology as that in the Sox2 mutants, including the truncated and excessive branching of CaP axons and the loss of MiP (Figure 3).

During the experiment, we found that in addition to the changes in motor neuron morphology, some MiP and RoP disappeared in the Sox2 mutants and morphants. To investigate whether this phenotype was caused by neural cell apoptosis, TUNEL assays were performed on the Sox2-deficient and control fish at 24 hpf. The results showed that the deficiency of Sox2 indeed caused cell apoptosis more seriously compared to the control group. However, the TUNEL signals were not colocalized with the GFP signal, indicating that the lack of motor neurons was not caused by neuron cell apoptosis (Supplementary Figure S3).

### Neuronal Cell Differentiation Was Affected by Loss Function of Sox2

In addition to alterations in motor neuron morphology, a large number of round GFP-positive cells that might be undifferentiated were found in Sox2 mutants and morphants (Figure 4). We discovered that many round neurons existed in the spinal cord at 24 hpf (data not shown) when both PMNs and SMNs do not completely generate. Although the number of the round neurons decreased rapidly with zebrafish development, it was also significantly higher in both Sox2 mutants and morphants than the control group at 48 and 72 hpf (Figure 4 and Supplementary Figure S2A).

### The Defects of Motor Neurons Could be Rescued by Overexpression of Sox2

To confirm that the motor neuron morphology defects were specifically caused by the Sox2 deficiency, we injected *in vitro*-synthesized Sox2 mRNA containing an intact open reading frame into one-cell stage Sox2 mutant embryos to test whether exogenous Sox2 could rescue the phenotype observed in the Sox2 mutants. Our results showed that the ratio of the abnormal zebrafish was significantly reduced after Sox2 mRNA injection, even if it was still higher than the control (Figures 5A,B). Moreover, the number of undifferentiated neuron cells in the spinal cord was decreased in the zebrafish injected with Sox2 mRNA, but still higher than that in the control zebrafish (Figure 5C). Similarly, the length of CaP was also partially rescued after the Sox2 mRNA injection (Figure 5D). In order to investigate whether Sox2 directly acts on motor neurons, we made a construct with the mnx1 driving Sox2 mRNA for the specific rescue experiment at 48 and 72 hpf. Unlike the ubiquitous rescue, motor neuron specific rescue failed to significantly increase the average length of CaP axons at 48 hpf. However, a significant improvement was observed at 72 hpf, but still significantly lower than in control embryos (Figure 5D).

### Transcriptomic Profiling of Sox2 Mutant and Wild-Type Zebrafish

**Figure 6:**
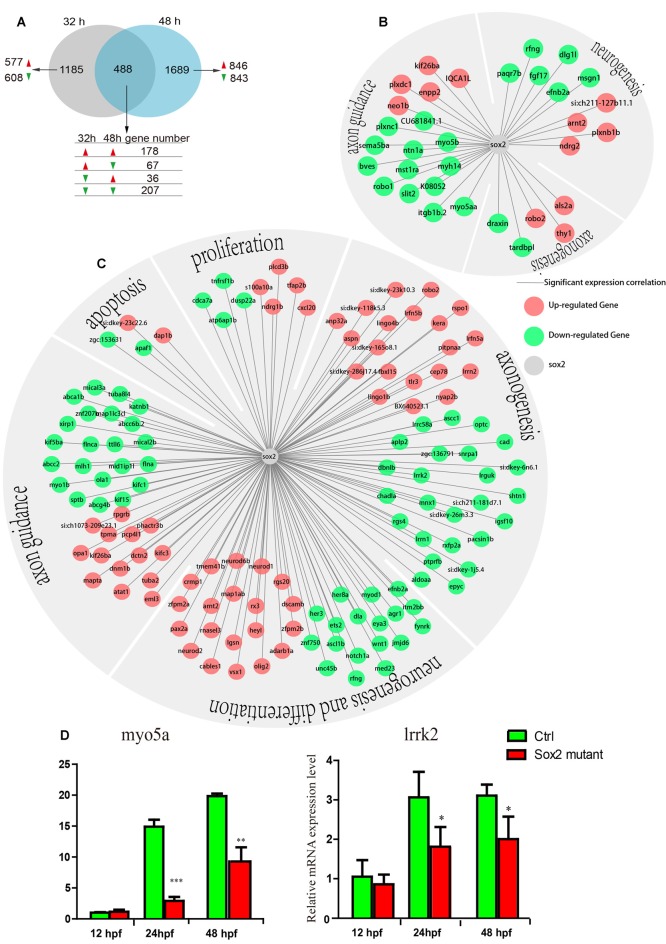
The results of transcriptomic profiling in Sox2 mutant and wild-type zebrafish. **(A)** The Venn chart in the control and Sox2 knockout treatment group. Differently continuous change types are displayed, red/green arrow means a gene up-/down-regulation at the time point. The expression correlation between Sox2 and differentially expressed genes (DEGs) at 32 h **(B)** and 48 h **(C)**; in the networks, the green node represents down-regulated DEGs and the red node represents up-regulated DEGs; moreover, all the DEGs were classified into different groups based on biological function. **(D)** Effects of Sox2 deficiency on the expressions of two motor neuron regulation-related genes. Experimental embryos were sampled at 12, 24, and 48 hpf (*n* = 12). Each bar represents the mean ± SD. Values with *, ** and *** above the bars are significantly different (*P* < 0.05, *P* < 0.01 and *P* < 0.001, respectively).

To get further insight into the mechanism by which Sox2 may influence motor neuron maturation, we performed RNA sequencing (RNA-seq) using the RNA sample from wild type and Sox2 mutant zebrafish at 32 and 48 hpf. The results revealed 1,673 differentially expressed genes (DEGs) that might be affected by the absence of Sox2 at 32 hpf with 822 up-regulated DEGs and 851 down-regulated DEGs (Figure 6A and Supplementary Tables S1, S2). Similarly, at 48 hpf, 2177 DEGs were found with 1060 up-regulated DEGs and another 1117 down-regulated DEGs. In addition, 178 related DEGs were up-regulated at both 32 and 48 hpf, while 207 DEGs were down-regulated. Moreover, 67 DEGs were up-regulated at 32 hpf but down-regulated at 48 hpf, while 36 DEGs were down-regulated at 32 hpf but up-regulated at 48 hpf. The correlation between DEGs and Sox2 expression was quantified by Pearson correlation coefficient. According to the GO and KEGG annotation, we classified the DEGs that have significant correlation with Sox2 into the different groups according to biological function: neurogenesis, axonogenesis, axon guidance, apoptosis, proliferation, and differentiation (Figures 6B,C).

RNA-seq results showed that the expression of *myo5a* and *lrrk2*, which contain the putative Sox2 binding sites, reduced in embryos with Sox2 loss of function (Figures 6B,C). We further examined the expression profiles of the two genes in different developmental stages of wild type and Sox2 mutant zebrafish embryos by qRT-PCR. Similar to the results of RNA-seq, the expression levels of *myo5a* and *lrrk2* were significantly decreased in Sox2 mutants at 24 and 48 hpf compared to that in the wild-type zebrafish (Figure 6D), which confirmed the quality of RNA-seq data.

## Discussion

Sox2 plays an important role in various biological processes of vertebrates. In addition to regulating the properties of stem cells, Sox2 is also involved in neuronal formation, differentiation, proliferation, and development (Zappone et al., 2000; Ogai et al., 2014). However, little is known about the role of Sox2 in motor neuron development. Currently, our data on zebrafish Sox2 expression and phenotype of Sox2 deficiency provide new insights into the role of Sox2 in motor neuron development.

This study used zebrafish as a model system to investigate the molecular and cellular mechanisms of motor neuron development. Firstly, we analyzed the expression of Sox2 in zebrafish larvae by *in situ* hybridization and found that it had a higher transcription level in the central nervous system such as the brain and spinal cord (Figure 1). During the metamorphosis of *Xenopus laevis*, high protein levels of Sox2 were detected in the ventricular layer cells of the spinal cord at R-stage (beginning of metamorphosis) by both immunofluorescence and Western blot, while the transcription levels of Sox2 did not significantly increase with development, indicating that the protein contents were regulated at post-transcription level (Muñoz et al., 2015). It has also been reported that Sox2 was expressed in the ependymal zone of the spinal cord in zebrafish and was induced at 1 day after spinal cord injury (Ogai et al., 2014). In newts, the migration of ependymal cells, which could further differentiate to motor neurons, to the ablation gap of the spinal cord was crucial for regeneration after spinal cord injury. This process provided a permissive condition for development of the axons to compensate for the loss of the motor neurons and reestablished the rostrocaudal continuity between the gap (Zukor et al., 2011; Goldshmit et al., 2012; Ogai et al., 2014; Muñoz et al., 2015). As far as we know, the spinal cord contains numerous motor neurons that project their axons to the terminal musculature during the development of the zebrafish embryo (Davis-Dusenbery et al., 2014). In view of the important role of Sox2 on the regeneration of the injured spinal cord, we want to know whether Sox2 is expressed in motor neurons and regulates the development of motor neurons. Therefore, we sorted the motor neurons from the *Tg(mnx1:GFP)^ml2^* transgenic zebrafish whose motor neurons were labeled by GFP *via* a flow cytometer. The RT-PCR showed that Sox2 was highly expressed in GFP-positive cells, suggesting that Sox2 might play an important role in the formation and development of motor neurons.

The results of the Sox2 mutants and morphants showed obvious motor neuron defects, including the reduction of MiP and the curtate but excessive branching of CaP axons. Normally, the CaPs are first observed in a ventral position and begin to sprout around 18 hpf. By 2 dpf, these motor neurons move to the typical dorsolateral location of the spinal cord and project their axons to the site between the notochord and the ventral edge of the axial muscles, and then the axons convert to dorsal muscles (Myers, 1985; Myers et al., 1986). Moreover, exuberant branches are formed around 3 dpf to further invade the myotome to form distributed neuromuscular synapses (Downes and Granato, 2004). Interestingly, with the depletion of Sox2, the axons of CaPs distinctly grew slowly, and the truncated axons could not project in the correct direction. Meanwhile, at 48 hpf, excess branches appeared in the axons of CaP in Sox2 mutant and morphant zebrafish relative to the control group. These results indicate that Sox2 plays important roles in the development of PMNs by regulating the axon morphology.

In addition to changes of the axon morphology, we also found that the deficiency of Sox2 could lead to the partial loss of MiP. We hypothesized that this phenotype might be caused by cell apoptosis. It has been reported that Sox2 was necessary for neuronal progenitor survival in the hippocampus and neural retina (Graham et al., 2003; Favaro et al., 2009). In Sox2-deficient mice oocysts, the activated caspase3, one kind of apoptotic cell death marker, stained cells were significantly more than that in the control group (Steevens et al., 2017). In zebrafish, more dying hair cells and support cells were found in the otic vesicle after Sox2 morpholino injection, suggesting that Sox2 was involved in the physiological regulation of the inner ear as a procursive factor (Millimaki et al., 2010; Steevens et al., 2017). In this study, we also found that the number of apoptotic cells in the trunk increased in the Sox2 mutants. However, these cells were not motor neurons nor were they located in the spinal cord, which suggested that the phenotype found in this study may not be associated with apoptosis. Similarly, it has been reported that although the deficiency of Sox2 could significantly increase the number of active Caspase3^+^ apoptotic cells in the subventricular zone of the Sox2 KO mice, the density of Caspase3^+^ Sox10^+^ apoptotic oligodendroglial cells was similar, indicating that Sox2 regulated the oligodendroglial progenitor cell population by another way but not cell survival (Zhang et al., 2018b). Taken together, these results indicate that Sox2 plays an important role in cell survival, but this function is cell type dependent.

It is well known that Sox2 encodes a transcription factor expressed in the CNS with the main role of maintaining the potency of neural stem cells to inhibit neuronal differentiation (Collignon et al., 1996; Graham et al., 2003). It has been reported that Sox2 was constitutively expressed in oligodendroglial progenitor cells to maintain proliferation of these neural cells and suppress the differentiation of oligodendrocytes (Shen et al., 2008; Pedre et al., 2011; Dai et al., 2015). However, in recent years, more and more studies have proposed contradictory results, that Sox2 positively controls the differentiation process of the neural stem cells (Hoffmann et al., 2014; Zhang et al., 2018a). Zhang et al. (2018b) reported that the density of newly differentiated oligodendrocytes of the brain subcortical white matter in the Sox2 KO mice was obviously lower than that in the control group, indicating that Sox2 positively regulates oligodendrocyte differentiation in the brain of murine. Hoffmann et al. (2014) also proved that Sox2 was required for the differentiation of oligodendrocytes in the embryonic spinal cord of oligodendroglial-specific Sox2 knockout mice. In the present study, we found that deficiency of Sox2 might impair the motor neuronal differentiation in the presence of many round neurons in the spinal cord. These results suggest that Sox2, as a transcriptional factor, also plays a crucial role in the cell differentiation of the CNS system.

As we know, a series of genes have been shown to be necessary for the formation and development of motor neurons. Our RNA-seq results showed that many DEGs between wild-type and Sox2 mutant zebrafish were involved in neurogenesis, axonogenesis, axon guidance, and differentiation. This result was coincident with the phenotype of Sox2 mutants, such as the truncated and excessively branched CaP axons, and increased the undifferentiation of neuron cells. In the rescue experiment, the recovery of the motor neuron was significantly only in ubiquitous rescue experiment but not motor neuron-specific rescue experiment at 48 hpf. This discrepancy between the two experiments indicated that Sox2 activity was likely to be required in the progenitors, prior to cell cycle exit, which indirectly affected the development of motor neuron. Such a role in progenitors is consistent with the fact that the RNA-seq data highlight a number of genes involved in neurogenesis and proliferation. Similarly, it has been reported that the formation of new neurons to restore the neuronal circuits after the spinal cord injury required the existence of Sox2 (Muñoz et al., 2015). In addition, the significant increase of axon length at 72 hpf in both ubiquitous and motor neuron-specific rescue experiments indicated that Sox2 activity was also required in motor neuron maturation, by controlling late events such as axon growth. Hence, the finding of multiple axon guidance genes in the RNA-seq was consistent with this view.

## Data Availability Statement

The raw data supporting the conclusions of this article will be made available by the authors, without undue reservation, to any qualified researcher.

## Ethics Statement

The animal study was reviewed and approved by Administration Committee of Experimental Animals, Jiangsu Province, China.

## Author Contributions

DL conceived the project. JG, SH, ZH, YH, XW, CW, JS, PQ, and XL performed most of the experiments. DL, JG, and GW analyzed the data and prepared the manuscript. All authors commented and approved the manuscript.

## Conflict of Interest

The authors declare that the research was conducted in the absence of any commercial or financial relationships that could be construed as a potential conflict of interest.

## References

[B1] Avilion A. A. Nicolis S. K. Pevny L. H. Perez L. Vivian N. Lovell-Badge R. (2003). Multipotent cell lineages in early mouse development depend on SOX2 function. Genes Dev. 17, 126–140. 10.1101/gad.224503 12514105 PMC195970

[B2] Babin P. J. Goizet C. Raldua D. (2014). Zebrafish models of human motor neuron diseases: advantages and limitations. Prog. Neurobiol. 118, 36–58. 10.1016/j.pneurobio.2014.03.001 24705136

[B3] Collignon J. Sockanathan S. Hacker A. Cohen-Tannoudji M. Norris D. Rastan S.. (1996). A comparison of the properties of Sox-3 with Sry and two related genes, Sox-1 and Sox-2. Development 122, 509–520. 8625802 10.1242/dev.122.2.509

[B4] Dai J. Bercury K. K. Ahrendsen J. T. Macklin W. B. (2015). Olig1 function is required for oligodendrocyte differentiation in the mouse brain. J. Neurosci. 35, 4386–4402. 10.1523/JNEUROSCI.4962-14.2015 25762682 PMC4461695

[B5] Davis-Dusenbery B. N. Williams L. A. Klim J. R. Eggan K. (2014). How to make spinal motor neurons. Development 141, 491–501. 10.1242/dev.097410 24449832

[B6] Downes G. B. Granato M. (2004). Acetylcholinesterase function is dispensable for sensory neurite growth but is critical for neuromuscular synapse stability. Dev. Biol. 270, 232–245. 10.1016/j.ydbio.2004.02.027 15136152

[B7] Eisen J. S. Pike S. H. Romancier B. (1990). An identified motoneuron with variable fates in embryonic zebrafish. J. Neurosci. 10, 34–43. 10.1523/JNEUROSCI.10-01-00034.1990 2299397 PMC6570358

[B8] Favaro R. Valotta M. Ferri A. L. Latorre E. Mariani J. Giachino C.. (2009). Hippocampal development and neural stem cell maintenance require Sox2-dependent regulation of Shh. Nat. Neurosci. 12, 1248–1256. 10.1038/nn.2397 19734891

[B9] Goldshmit Y. Sztal T. E. Jusuf P. R. Hall T. E. Nguyen-Chi M. Currie P. D. (2012). Fgf-dependent glial cell bridges facilitate spinal cord regeneration in zebrafish. J. Neurosci. 32, 7477–7492. 10.1523/JNEUROSCI.0758-12.2012 22649227 PMC6703582

[B10] Gong J. Wang X. Zhu C. Dong X. Zhang Q. Wang X.. (2017). Insm1a regulates motor neuron development in zebrafish. Front. Mol. Neurosci. 10:274. 10.3389/fnmol.2017.00274 28894416 PMC5581358

[B11] Gou Y. Vemaraju S. Sweet E. M. Kwon H.-J. Riley B. B. (2018). Sox2 and sox3 play unique roles in development of hair cells and neurons in the zebrafish inner ear. Dev. Biol. 435, 73–83. 10.1016/j.ydbio.2018.01.010 29355523 PMC5818298

[B12] Graham V. Khudyakov J. Ellis P. Pevny L. (2003). SOX2 functions to maintain neural progenitor identity. Neuron 39, 749–765. 10.1016/s0896-6273(03)00497-5 12948443

[B13] Haramati S. Chapnik E. Sztainberg Y. Eilam R. Zwang R. Gershoni N.. (2010). miRNA malfunction causes spinal motor neuron disease. Proc. Natl. Acad. Sci. U S A 107, 13111–13116. 10.1073/pnas.1006151107 20616011 PMC2919953

[B14] Helmbacher F. Schneider-Maunoury S. Topilko P. Tiret L. Charnay P. (2000). Targeting of the EphA4 tyrosine kinase receptor affects dorsal/ventral pathfinding of limb motor axons. Development 127, 3313–3324. 10887087 10.1242/dev.127.15.3313

[B15] Hoffmann S. A. Hos D. Küspert M. Lang R. A. Lovell-Badge R. Wegner M.. (2014). Stem cell factor Sox2 and its close relative Sox3 have differentiation functions in oligodendrocytes. Development 141, 39–50. 10.1242/dev.098418 24257626 PMC3865748

[B16] Huang Y. Wang X. Wang X. Xu M. Liu M. Liu D. (2013). Nonmuscle myosin II-B (myh10) expression analysis during zebrafish embryonic development. Gene Expr. Patterns 13, 265–270. 10.1016/j.gep.2013.04.005 23665442

[B17] Hutchinson S. A. Cheesman S. E. Hale L. A. Boone J. Q. Eisen J. S. (2007). Nkx6 proteins specify one zebrafish primary motoneuron subtype by regulating late islet1 expression. Development 134, 1671–1677. 10.1242/dev.02826 17376808 PMC2586877

[B18] Kamachi Y. Uchikawa M. Kondoh H. (2000). Pairing SOX off: with partners in the regulation of embryonic development. Trends Genet. 16, 182–187. 10.1016/s0168-9525(99)01955-1 10729834

[B19] Lewis K. E. Eisen J. S. (2003). From cells to circuits: development of the zebrafish spinal cord. Prog. Neurobiol. 69, 419–449. 10.1016/s0301-0082(03)00052-2 12880634

[B20] McWhorter M. L. Monani U. R. Burghes A. H. Beattie C. E. (2003). Knockdown of the survival motor neuron (Smn) protein in zebrafish causes defects in motor axon outgrowth and pathfinding. J. Cell Biol. 162, 919–932. 10.1083/jcb.200303168 12952942 PMC1761110

[B21] Messersmith E. K. Leonardo E. D. Shatz C. J. Tessier-Lavigne M. Goodman C. S. Kolodkin A. L. (1995). Sernaphorin III can function as a selective chemorepellent to pattern sensory projections in the spinal cord. Neuron 14, 949–959. 10.1016/0896-6273(95)90333-x 7748562

[B22] Millimaki B. B. Sweet E. M. Riley B. B. (2010). Sox2 is required for maintenance and regeneration, but not initial development, of hair cells in the zebrafish inner ear. Dev. Biol. 338, 262–269. 10.1016/j.ydbio.2009.12.011 20025865 PMC2815045

[B23] Moreno R. L. Ribera A. B. (2009). Zebrafish motor neuron subtypes differ electrically prior to axonal outgrowth. J. Neurophysiol. 102, 2477–2484. 10.1152/jn.00446.2009 19692510 PMC2775388

[B24] Muñoz R. Edwards-Faret G. Moreno M. Zuñiga N. Cline H. Larraín J. (2015). Regeneration of *Xenopus laevis* spinal cord requires Sox2/3 expressing cells. Dev. Biol. 408, 229–243. 10.1016/j.ydbio.2015.03.009 25797152 PMC4826040

[B25] Myers P. Z. (1985). Spinal motoneurons of the larval zebrafish. J. Comp. Neurol. 236, 555–561. 10.1002/cne.902360411 4056102

[B26] Myers P. Z. Eisen J. S. Westerfield M. (1986). Development and axonal outgrowth of identified motoneurons in the zebrafish. J. Neurosci. 6, 2278–2289. 10.1523/JNEUROSCI.06-08-02278.1986 3746410 PMC6568750

[B27] Ogai K. Nakatani K. Hisano S. Sugitani K. Koriyama Y. Kato S. (2014). Function of Sox2 in ependymal cells of lesioned spinal cords in adult zebrafish. Neurosci. Res. 88, 84–87. 10.1016/j.neures.2014.07.010 25150399

[B28] Park H.-C. Mehta A. Richardson J. S. Appel B. (2002). olig2 is required for zebrafish primary motor neuron and oligodendrocyte development. Dev. Biol. 248, 356–368. 10.1006/dbio.2002.0738 12167410

[B29] Pedre X. Mastronardi F. Bruck W. López-Rodas G. Kuhlmann T. Casaccia P. (2011). Changed histone acetylation patterns in normal-appearing white matter and early multiple sclerosis lesions. J. Neurosci. 31, 3435–3445. 10.1523/JNEUROSCI.4507-10.2011 21368055 PMC3081530

[B30] Puligilla C. Dabdoub A. Brenowitz S. D. Kelley M. W. (2010). Sox2 induces neuronal formation in the developing mammalian cochlea. J. Neurosci. 30, 714–722. 10.1523/JNEUROSCI.3852-09.2010 20071536 PMC2835399

[B31] Rodino-Klapac L. R. Beattie C. E. (2004). Zebrafish topped is required for ventral motor axon guidance. Dev. Biol. 273, 308–320. 10.1016/j.ydbio.2004.06.007 15328015

[B32] Shen S. Sandoval J. Swiss V. A. Li J. Dupree J. Franklin R. J.. (2008). Age-dependent epigenetic control of differentiation inhibitors is critical for remyelination efficiency. Nat. Neurosci. 11, 1024–1034. 10.3410/f.1124012.581123 19160500 PMC2656679

[B33] Shirasaki R. Pfaff S. L. (2002). Transcriptional codes and the control of neuronal identity. Annu. Rev. Neurosci. 25, 251–281. 10.1146/annurev.neuro.25.112701.142916 12052910

[B34] Steevens A. R. Sookiasian D. L. Glatzer J. C. Kiernan A. E. (2017). SOX2 is required for inner ear neurogenesis. Sci. Rep. 7:4086. 10.1038/s41598-017-04315-2 28642583 PMC5481345

[B35] Takahashi K. Yamanaka S. (2006). Induction of pluripotent stem cells from mouse embryonic and adult fibroblast cultures by defined factors. Cell 126, 663–676. 10.1016/j.cell.2006.07.024 16904174

[B36] Wang X. Ling C. C. Li L. Qin Y. Qi J. Liu X.. (2016). MicroRNA-10a/10b represses a novel target gene mib1 to regulate angiogenesis. Cardiovasc. Res. 110, 140–150. 10.1093/cvr/cvw023 26825552

[B37] Xu M. Liu D. Dong Z. Wang X. Wang X. Liu Y.. (2014). Kinesin-12 influences axonal growth during zebrafish neural development. Cytoskeleton 71, 555–563. 10.1002/cm.21193 25250533 PMC4236235

[B38] Zappone M. V. Galli R. Catena R. Meani N. De Biasi S. Mattei E.. (2000). Sox2 regulatory sequences direct expression of a (beta)-geo transgene to telencephalic neural stem cells and precursors of the mouse embryo, revealing regionalization of gene expression in CNS stem cells. Development 127, 2367–2382. 10804179 10.1242/dev.127.11.2367

[B39] Zelenchuk T. A. Brusés J. L. (2011). *In vivo* labeling of zebrafish motor neurons using an mnx1 enhancer and Gal4/UAS. Genesis 49, 546–554. 10.1002/dvg.20766 21538811 PMC3642388

[B40] Zhang S. Rasai A. Wang Y. Xu J. Bannerman P. Erol D.. (2018a). The stem cell factor sox2 is a positive timer of oligodendrocyte development in the postnatal murine spinal cord. Mol. Neurobiol. 55, 9001–9015. 10.1007/s12035-018-1035-7 29623612 PMC6173662

[B41] Zhang S. Zhu X. Gui X. Croteau C. Song L. Xu J.. (2018b). Sox2 is essential for oligodendroglial proliferation and differentiation during postnatal brain myelination and CNS remyelination. J. Neurosci. 38, 1802–1820. 10.1523/JNEUROSCI.1291-17.2018 29335358 PMC5815459

[B42] Zukor K. A. Kent D. T. Odelberg S. J. (2011). Meningeal cells and glia establish a permissive environment for axon regeneration after spinal cord injury in newts. Neural Dev. 6:1. 10.1186/1749-8104-6-1 21205291 PMC3025934

