# Supplementary material for: The Requirement of Sox2 for the Spinal Cord Motor Neuron Development of Zebrafish
Source: Front Mol Neurosci. 2020 Mar 27;13:34. doi: 10.3389/fnmol.2020.00034 (PMC7135881; doi:10.3389/fnmol.2020.00034)
Supplement: Supplementary file 3 [file Data_Sheet_1.docx]

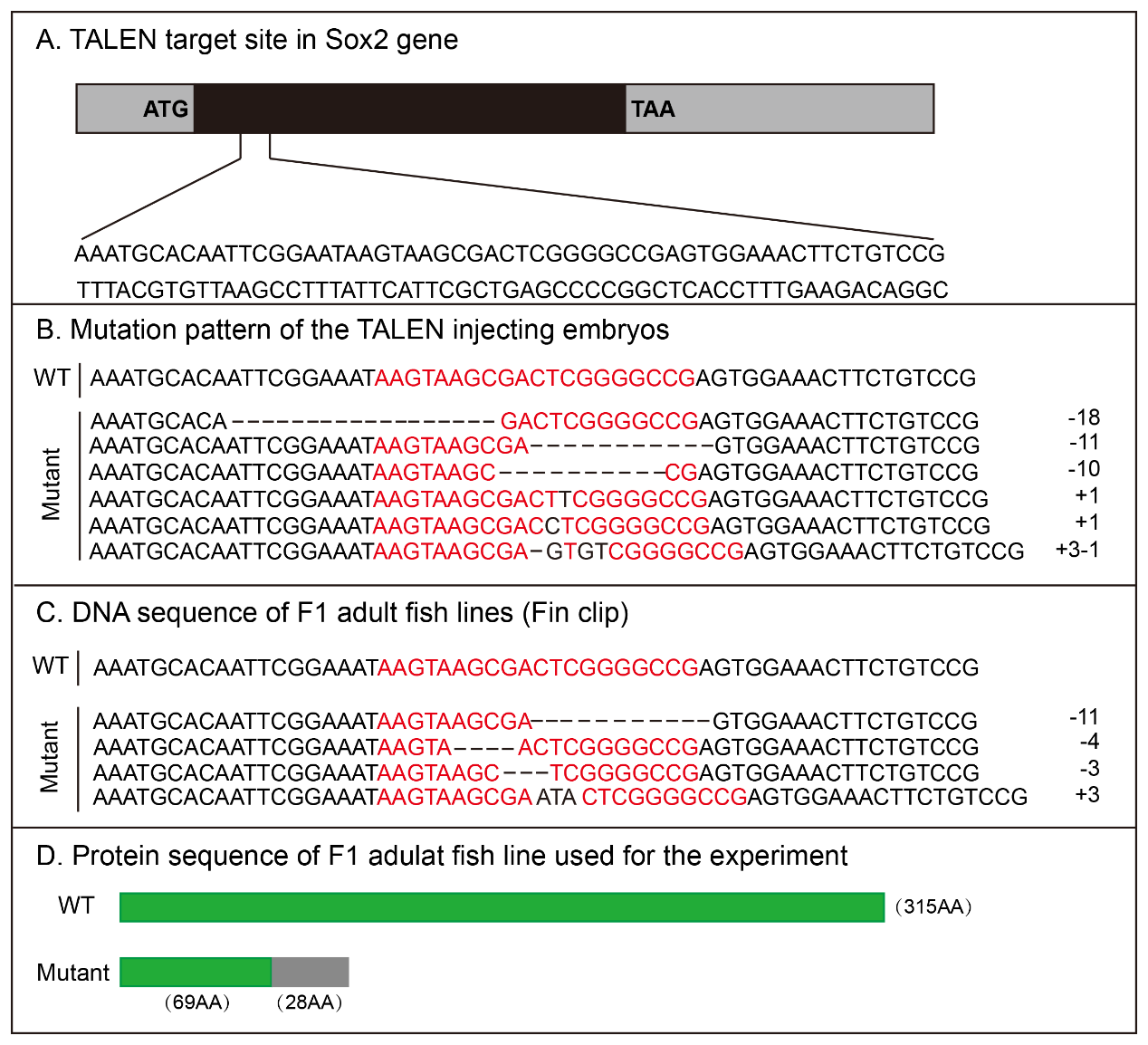


**Supplementary Figure 1. Generation of zebrafish Sox2 mutant using TALEN system.** A. Schematic diagram showing TALEN targeting site. B. Mutation pattern of G0 embryos. Number of insertion or deletion base pairs showed on the right. C. Mutation pattern of F1. D. Schematic diagram showing the predicted proteins encoded by the mutated alleles (-4 used in this study). The mutants are reading frame shift mutations that result in truncated proteins. The gray rectangles indicate the wrong coded amino acid sequences.


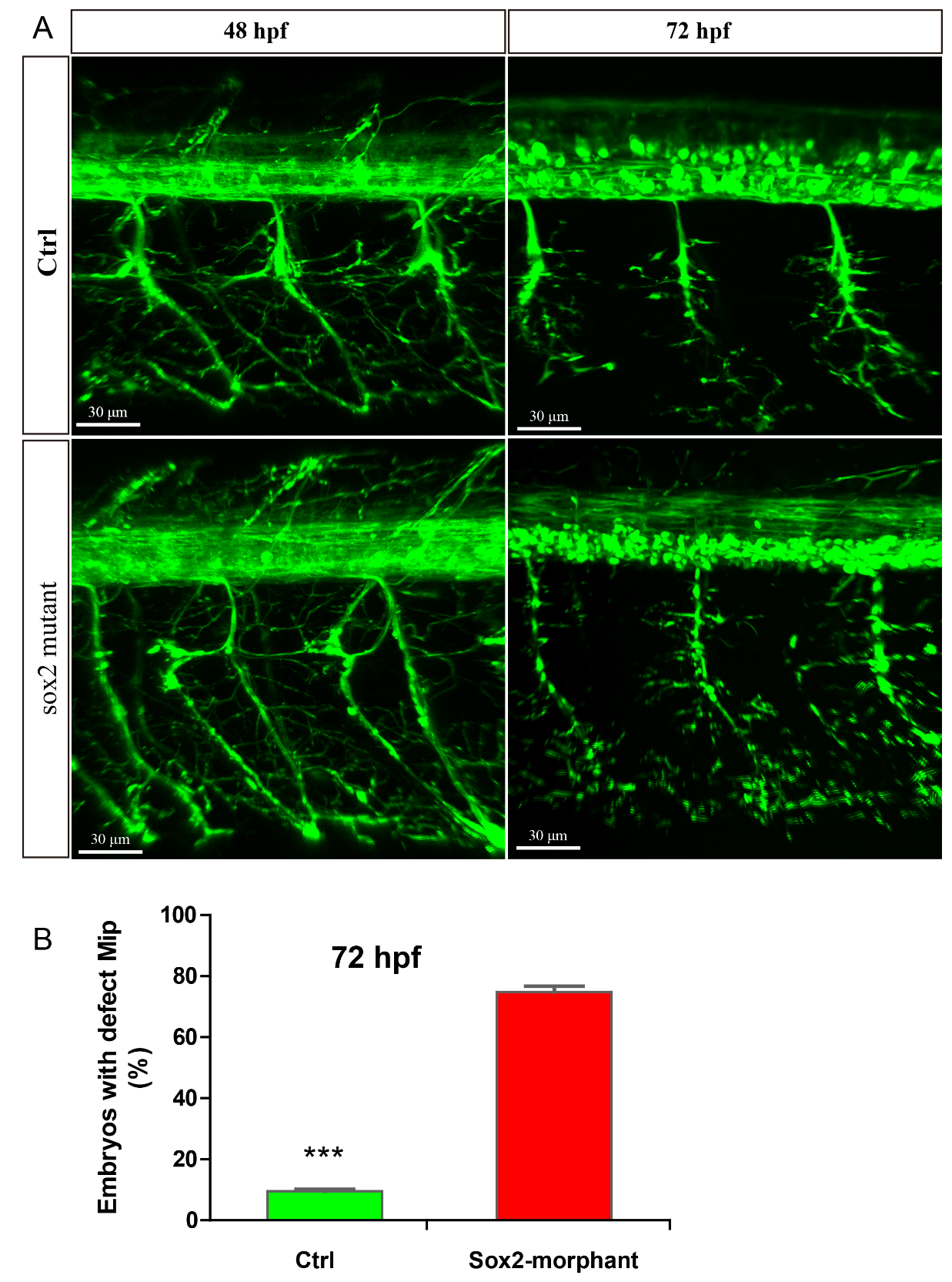


**Supplementary Figure 2.** Primary motor neuron morphogenesis defects in the Sox2 knockout zebrafish. A. Confocal imaging analysis (40x objective) of primary motor neurons in control and Sox2 knockout groups at 48 and 72 hpf. Scale bar= 30μm. B. Quantification of zebrafish embryos with abnormal PMNs. Each bar represents the mean ± SD. Value with ***above the bars is significantly different (P<0.01 and P<0.001, respectively).


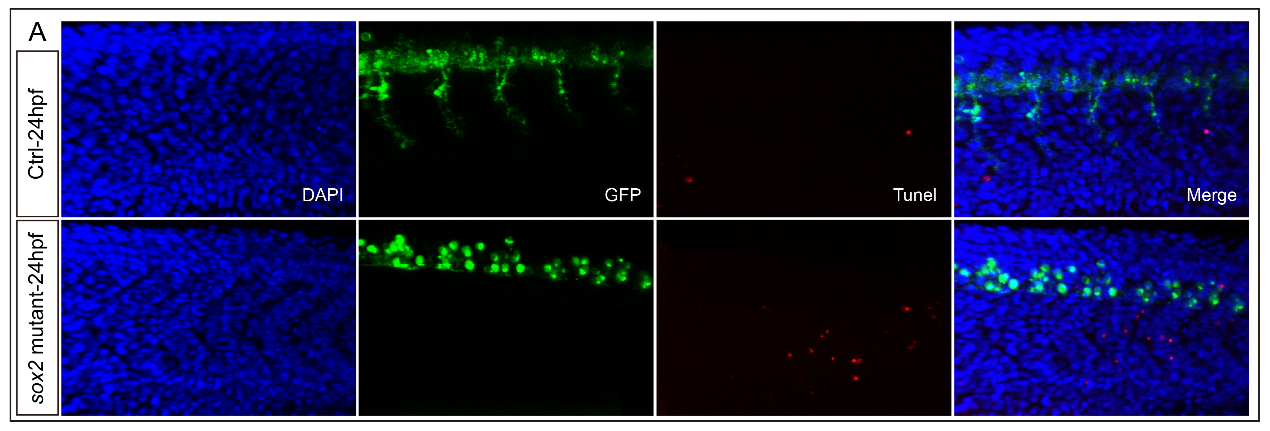


**Supplementary Figure 3. Effect of Sox2 on apoptosis.** A and A’. The results of DAPI staining in control group, and Sox2 mutant group at 24 hpf; B and B’. Confocal imaging results of primary motor neurons. C and C’. The results of TUNEL. Scale bar= 50 μm.

**Supplementary Table S1: The list of significant different expressed genes between the control fish and Sox2 mutants at 32 hpf.**

**Supplementary Table S2: The list of significant different expressed genes between the control fish and Sox2 mutants at 48 hpf.**
